# Evolution of eye development in the darkness of caves: adaptation, drift, or both?

**DOI:** 10.1186/2041-9139-4-26

**Published:** 2013-09-30

**Authors:** Sylvie Rétaux, Didier Casane

**Affiliations:** 1 DECA group, Neurobiology & Development Laboratory, CNRS, Gif sur Yvette, France; 2 LEGS, CNRS, Gif sur Yvette and Université Paris Diderot, Sorbonne Paris Cité, France

**Keywords:** Astyanax, lens, retina, transcriptome, population biology

## Abstract

Animals inhabiting the darkness of caves are generally blind and de-pigmented, regardless of the phylum they belong to. Survival in this environment is an enormous challenge, the most obvious being to find food and mates without the help of vision, and the loss of eyes in cave animals is often accompanied by an enhancement of other sensory apparatuses. Here we review the recent literature describing developmental biology and molecular evolution studies in order to discuss the evolutionary mechanisms underlying adaptation to life in the dark. We conclude that both genetic drift (neutral hypothesis) and direct and indirect selection (selective hypothesis) occurred together during the loss of eyes in cave animals. We also identify some future directions of research to better understand adaptation to total darkness, for which integrative analyses relying on evo-devo approaches associated with thorough ecological and population genomic studies should shed some light.

## Review

### The cave environment

Water- and air-filled cavities are abundant in all continents but Antarctica. North America and Eurasia are especially rich in cave-bearing rocks. Actually, more than 94% of the world’s unfrozen freshwater is stored underground. For example, in the US alone nearly 50,000 caves are known [1], and it has been estimated that there are 521,000 km^3^ of subsurface cavities, most of these containing water. This is a huge underground world that is poorly known, in particular the diversity of life it contains. Among the physicochemical properties of cave environments, the most striking is the complete absence of sunlight, which means no photosynthesis and therefore the absence of primary producers (plants, algae, and bacteria) relying on it. Although certain caves hosting large bats colonies are food-rich (guano-rich), most cave communities rely on food transported in from the surface. In the absence of autotrophy, the amount and variety of resources are usually low and irregular. In some cases, spring flooding may be important as seasonal input bringing animal and vegetal debris and sediments into caves. Nevertheless the amplitude of variation of many environmental parameters, in particular temperature, is much less than that of the surface habitats [2].

### The diversity of cave animals

Many animals are temporary visitors of caves, but here we will focus on obligate and permanent residents. These animals are called troglobionts (aquatic species are sometimes called stygobionts). The absence of light has major effects on these organisms. Food and mate finding as well as predator avoidance must be accomplished without vision. Color patterns that are often involved in intra-specific recognition and camouflage are useless, just like the visual system (the eyes, the connecting nerves through to the visual brain areas and other parts of the brain). Cave environments generally allow the maintenance of small populations as the result of food scarcity and a lower biodiversity than can reduce interspecific predation, if any. It thus has a strong impact on life history traits such as the reproductive lifespan, aging, number and size of offspring [2]. Troglobionts often show a combination of regressive characters (e.g., loss of eyes and pigmentation) and constructive characters (e.g., enhanced sensory structures not based on light sensing, longer lifespan, larger eggs, lower metabolism rate) that evolved independently in different lineages in relation with the cave environment. These evolutionary convergences allow identifying some evolutionary trends associated with this environment [3-5].

There are probably tens of thousands of troglobiont species. Actually the number of known species has increased very quickly since their initial discovery. For example, the number of known fish species has tripled in the last 30 years (from 43 to 150 between 1980 and 2010) [6]. These actinopterygian fishes, which belong to ten orders, together with a couple of amphibian species represent the troglobiont vertebrates. Among protostomes, more than 50 orders belonging to the phyla Platyhelminthes, Annelida, Mollusca and Arthropoda contain troglobiont species [2,5]. The wide phylogenetic distribution of cave animals indicates that the adaptation to caves occurred independently many times in different phyla. The observation of closely related epigean species (or populations) indicates that some troglobiont species are of recent origin. Some very ancient groups of troglobiont species are also known, such as the crustacean class Remipedia containing only stygobiontic species [2,5]. These observations suggest that cave animals are neither Darwin’s ‘wrecks of ancient life’ nor necessarily ‘evolutionary dead ends’ in the short term [7]. Nevertheless, the relative roles of adaptation and genetic drift in the evolution of the convergent traits observed in these organisms have been much debated.

### Ecology and evolutionary developmental biology of cave animals: EcoEvoDevo

After a period of strong criticisms, adaptation has recently come back as a central issue in evolutionary biology [8]. In 1966, George C. Williams published an influential book, “Adaptation and Natural Selection,” in which he discredited the usage of a naive adaptationist reasoning. He pointed out that adaptation is “a special and onerous concept that should only be used where it is really necessary” [9]. In 1977, Stephen J. Gould and Richard C. Lewontin published their famous paper “The Sprandrels of San Marco and the Panglossian Paradigm: A Critique of the Adaptationist Programme” in which they criticized the adaptationist hypothesis that viewed all features of organisms as *a priori* optimal features produced by natural selection specifically for their current function. They demanded that evolutionary biologists consider alternatives, and they emphasized the notion that “organisms must be analyzed as integrated wholes, with Baupläne so constrained by phyletic heritage, pathways of development, and general architecture” [10]. In parallel, the observation of a huge polymorphism at the DNA level led to the proposal of the neutral theory of molecular evolution, which states that random drift is a major mechanism of genome evolution [11]. The study of adaptation is thus now based on a renewed conceptual framework that takes into account demographic effects (genetic drift, migration) and the complexity of the responses to selection due in particular to the pleiotropy of many genes. Fortunately, new experimental approaches such as genome-wide sequencing and new statistical tools allow tackling the complexity of the evolutionary process [12,13]. It is noteworthy that adaptation actually has various definitions in different domains of biology. Here, in the context of adaptation to a special lightless environment, we use a narrow definition to discuss issues about genetic changes: adaptation is the evolutionary process whereby an organism becomes better able to live in its habitat. Non-anthropogenic catastrophic changes of habitat often correspond to populations trapped in exceptional or “extreme” environments where they are ill-adapted. Often these populations become extinct, but in a few cases they adapt and even flourish. Among the rapid switches to environments that lead to spectacular adaptation, caves are an especially relevant ecosystem.

### Drift or adaptation? A historical perspective

In 1842, *Amblyopsis spelaea*, which lives in Mammoth Cave (Kentucky, USA) was the first subterranean fish species formally described. However, the most famous cave animal—and also the first described, by Laurenti in 1768—is the salamander *Proteus anguinus*, which lives in karstic caves of Southeastern Europe. Darwin saw these two subterranean animals as examples of eyelessness and loss of structure in general. For him, the explanation was a straightforward Lamarckian one, and one that did not involve adaptation and the struggle for existence. He wrote: “It is well known that several animals which inhabit caves of Carniola [*P. anguinus*] and Kentucky [Amblyopsid fishes] are blind…As it is difficult to imagine that eyes, though useless, could be in any way injurious to animals living in darkness, their loss may be attributed to disuse” [14]. Although this Lamarckian theory should have been quickly discredited, it happened only during the second part of the twentieth century, when Wilkens [15] refined genetic analyses of this “regressive” evolution in the fish *Astyanax mexicanus* (see [5] for a detailed historical perspective). Wilkens proposed that eye and pigment loss was almost entirely the result of the accumulation of morphologically reducing, selectively neutral mutations. In the early 2000s, Jeffery challenged this neutral theory of regressive evolution and emphasized the importance of selection on constructive traits and the indirect effects on regressive traits through the spread of mutations in pleiotropic genes [16].

At the molecular level, neutral and selective hypotheses are not mutually exclusive. Indeed, in the framework of the nearly neutral theory of molecular evolution [17], one expects that most mutations arising in a genome are neutral or slightly deleterious, some are highly deleterious, and a few are advantageous.

First of all the mutation rate can diverge in cave and epigean populations. One reason is the fixation of mutations in proteins involved in the replication and reparation of the DNA that can change the accuracy of these processes. We expect that a higher rate of mutation can evolve in caves because of a higher rate of fixation of slightly deleterious mutations (see below) that reduce the efficiency of the proteins involved in DNA replication and reparation. The main factors controlling the mutation rate are the effective genome size (the length of the DNA sequence under selection, not relevant when comparing hypogean and epigean populations in the same species) and the effective population size, which limits the efficiency of selection toward ever-lower mutation rates [18-23]. The other reason is differences in the impact of some mutagenic factors such as chemical substances and UV radiations in these two environments [20].

**Figure 1:**
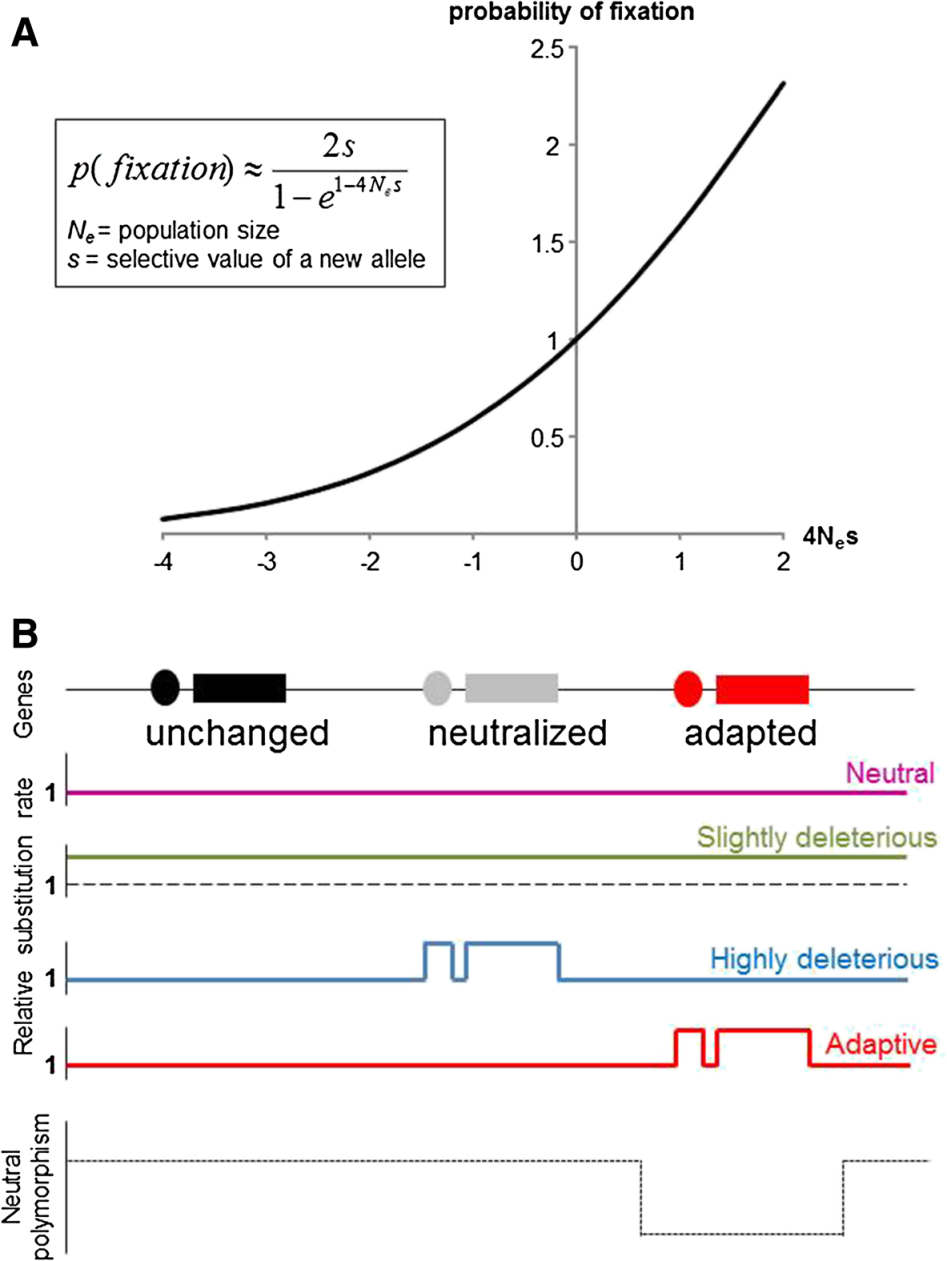
**Effect of population size and environmental modifications on the substitution rate (rate of fixation of mutations in the whole population). A** Relative probability of fixation of a mutation according to the population size (N_e_) and its selective value (s) [24,25]. s = 0 => neutral; s < 0 => deleterious; s > 0 => advantageous. Y axis scale: probability relative to the probability of fixation of a neutral mutation. Kimura suggested a simple rule of thumb: if |s| < < 1/4N_e_ selection is weak and genetic drift dictates allele fixation; otherwise, selection dominates. A slightly deleterious mutation with s = -0.001 has a high probability to reach fixation in a population of size 250 (when 4N_e_s = -1, relative probability = 0.58, i.e., about half the probability of fixation of a neutral mutation), whereas it has a very low probability to be maintained in a population of size 2,500 (when 4N_e_s = -10, relative probability = 0.00045). **B** Relative substitution rate of mutations according to the selective values and constraint changes on genome after a shift in cave environment. The constraints on coding sequences (*rectangles*) and their regulatory sequences (*ovals*) can be “unchanged,” lifted if a protein has no function (“neutralized”), or “changed” due to adaptation to this new environment. Y axis scale: The substitution rate relative to the substitution rate in a larger population and before the environmental shift. Neutral mutations (*pink*) should accumulate at the same rate; slightly deleterious mutations (*green*) should accumulate at a higher rate in all sequences because they behave as neutral mutations in a small population; previously highly deleterious mutations (*blue*) could accumulate in “neutralized” sequences; adaptive mutations (*red*) would accumulate in sequences involved in the adaptation to the environmental shift. In parallel, there is a reduction or elimination of polymorphism (*black dotted lines*) in the neighboring DNA of a mutation under recent and strong positive selection (selective sweep due to genetic hitchhiking).

The substitution rate (the rate of fixation of a mutation in a population) of neutral mutation is independent of the population size [11]. The substitution rate of slightly deleterious mutations depends on the population size. Indeed, in small populations, slightly deleterious mutations behave as neutral mutations because selection is less efficient relative to genetic drift [24,25] (Figure 1A). Most cave-dwelling species are found in very restricted areas, and their population sizes are usually small [26-29]. When closely related species or populations have been identified in some large areas, they often correspond to independent adaptation to this environment. These observations show the limited dispersal possibility of most animals living in hypogean environment [30-41]. We thus expect an increase in the substitution rate of slightly deleterious mutations in small hypogean populations relative to epigean populations. Moreover, highly deleterious mutations in surface populations can be neutral in cave populations because they occurred in genes that are no longer under selection in this environment. In addition, some mutations can reach fixation because they confer a better fitness. Disentangling the combined effects of genetic drift, purifying and adaptive selection is a major challenge to better understand the evolution of troglobionts (Figure 1B).

At the genome-wide scale, we expect a global increase in the substitution rate in troglobionts due to fixation of slightly deleterious mutations that would be eliminated in large populations, but behave as effectively neutral in such small populations. We would also expect a local increase in the substitution rate in “neutralized” genes (i.e., not under selection in caves) and a local increase in the substitution rate in adaptive genes (i.e., fixation of mutations conferring adaptive value in caves). Analyzing the molecular evolution of different gene networks that are implicated to varying extents in the phenotypic changes observed in troglobionts could allow the identification of relaxed selection on some genes. Some phenotypes, such as eye regression and pigment reduction, have evolved and/or persisted in some cave populations despite gene flow from surface populations [32,39], and this observation alone would be in conflict with any theory for the origin of these phenotypes that is not based at least in part on natural selection. Population genomic approaches, such as the detection of selective sweep, in particular in the context of gene flow, should allow identifying the fixation of adaptive mutations [42].

Interestingly, Culver and colleagues, working on the cave amphipod *Gammarus minus*, concluded that cave characters can be divided into ‘regressive’ and ‘constructive’ traits and that evolutionary mechanisms likely differ depending upon the category. They argued that regressive traits (eye size) evolve faster, reasoning they arise through additive effects of drift and selection. Conversely, constructive traits (antennal size) evolve slower since they arise “merely” through direct selection [4]. In the cavefish *Astyanax mexicanus*, on the other hand, genetic studies suggest that eyes and pigmentation regressed through different mechanisms [43]. Cave alleles at every eye or lens QTL cause size reductions, consistent with evolution by natural selection but not with drift. Conversely, QTL polarities for the melanophore number are mixed, consistent with genetic drift. Below, we take the case of the loss of eyes in cave animals to illustrate the mechanisms that have been deciphered so far to explain morphological evolution in caves, through the different but complementary approaches of evo-devo and comparative transcriptomics.

### The loss of eyes: insights from developmental biology

#### ***The eyes first develop and then regress in cave animals***

As stated above, cave animals from all phyla are eyeless or have strongly reduced eyes. One puzzling fact however is that all the independently evolved vertebrate cave species that have been investigated so far at embryonic stages have shown normal initial eye development: an optic cup and a lens placode form, followed by differentiated retinal layers and a lens mass, respectively. But rapidly after these early stages of morphogenesis, things start going wrong, and interestingly, they seem to follow the same type of cellular path in various animals.

The cave salamander *Proteus anguinus* (an amphibian) has been studied for two centuries as an emblematic animal and only real cave-dwelling vertebrate in Europe. As stated by Durand, “initial eye development is perfectly normal” [44]. But then growth slows down, the cornea involutes, and the lens undergoes severe lytic processes, which ultimately result in an eye that is strongly reduced and sunken into the orbits.

The underground-living naked mole rat *Heterocephalus glaber* (a mammal) has very small eyes and poor image-forming visual abilities, if any, although rods and S-opsin-expressing cones are present in its retina [45]. Its eyes are described as microphtalmic, with structural defects in the cornea, iris, lens and retina, and with a proposed central role for the lens in the abnormal development of the eyes after birth [46].

We currently know about 150 fish species worldwide that have evolved to live in cave habitats [6]. Some have been investigated for eye development. In the cave catfish of the genus *Rhamdia* (a siluriform), the eyes are smaller than those of surface *Rhamdia* from the very beginning, necrotic processes arise in the lens, and secondary lens fibers do not elongate properly [47]. The Somalian cavefish *Phreatichthys andruzzii* (a cypriniform) is often considered anophtalmic at the adult stage, yet its embryos develop normal eyes with a retina, lens and cornea until 36 h post-fecundation (hpf). Developmental arrest and complete degeneration then occur very fast within a month, starting with apoptotic processes in the lens together with interrupted differentiation and autolytic processes in the retina [48]. In the “double extremophile” *Poecilia mexicana* (a cyprinodontiform), which not only lives in the dark but also in hydrogen sulfide-rich pools, eye growth stops after initial organ formation, and the adults have small eyes [49]. Recently, morphology and transcriptomic gene expression studies have been performed in cave-dwelling *Sinocyclocheilus anophtalmus* (another cypriniform) with small internal eyes [50]. In this species, evolved retinal reduction occurs in a lens-independent fashion by the reduced proliferation and downregulation of transcriptional factors shown to have direct roles in retinal development and maintenance, including cone-rod homeobox (crx) and Wnt pathway members.

**Figure 2:**
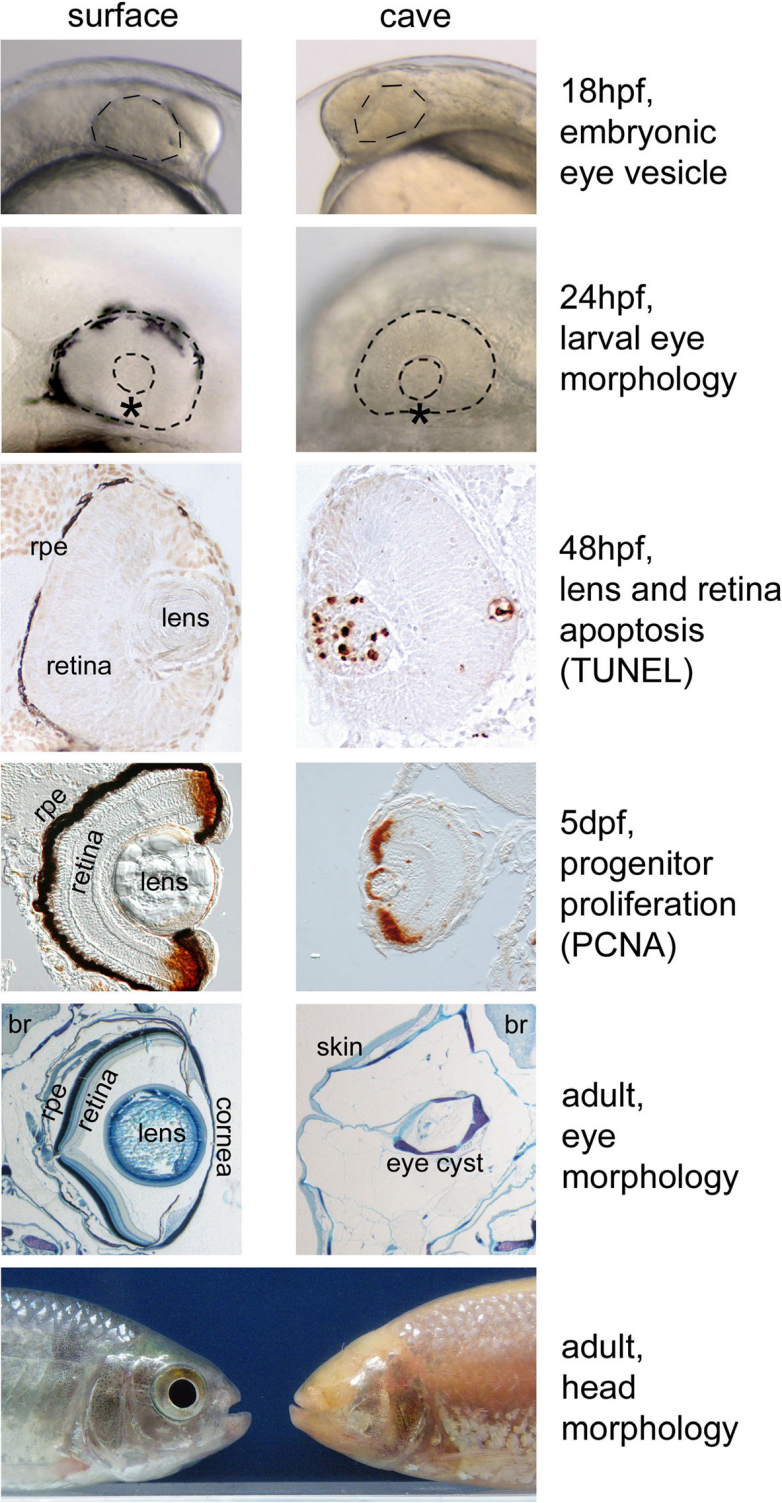
**Comparing *Astyanax mexicanus *surface fish and cavefish eye development and degeneration.** Representative stages and events of eye development are shown for surface fish (*left column*) and Pachón cavefish (*right column*). The asterisk on 24-hpf photographs represents the ventral quadrant of the retina, which is missing in cavefish. For each line, the surface fish and the cavefish pictures are at the same scale. *hpf* hours post-fertilization; *dpf* days post-fertilization; *rpe* retinal pigmented epithelium; *br* brain.

Finally, the fish species in which these developmental events have been best studied is *Astyanax mexicanus* (a characiform). In cave *Astyanax*, 1 day after fecundation, a “normal” eye has formed that is only slightly smaller than in *Astyanax* surface fish [51,52]. But soon the lens enters apoptosis, retinal cells are born and die at a high rate, growth stops, and in adults only a residual cyst sunken into the orbits and covered by skin can be observed [15,53-56] (Figure 2). Of note, in cave *Astyanax* a contribution from both the lens (which is apoptotic and does not send correct signals to the retina) and the retinal pigmented epithelium (from which signaling would be absent or non-functional) has been suggested to explain arrested retinal growth [57].

Thus, in these many and completely independent cases of regressive evolution in cave vertebrates, the two keywords for eye developmental degeneration seem to be: lens cell death and arrested growth. We believe that the genetic, cellular and molecular mechanisms by which cave animals lose their eyes by degeneration after initial development reflect and are highly informative concerning the evolutionary processes at work during adaptation to life in the dark. A prediction would be the following: (1) if the loss of eyes is due to the accumulation of loss of function mutations in eye developmental genes, then that would argue either in favor of drift and neutral evolution or in favor of direct selection (if it is advantageous to be eyeless in the dark); (2) if the loss of eyes is due to pleiotropic regulatory changes in developmental events that otherwise modulate other anatomical features that are advantageous to live in the dark, then the mechanism would be indirect selection. Of note, these mechanisms are not necessarily mutually exclusive.

#### ***Developmental mechanisms for eye loss in Astyanax mexicanus***

The *Astyanax* model system [26], with its different and partly independently evolved natural populations of cavefish [32], its surface populations that belong to the same species and therefore interbred with cave animals, and its relative ease in husbandry that allows obtaining hundreds of embryos [51], is ideal to investigate the evolutionary developmental mechanisms of eye loss [58-60].

There are in fact two clear defects in *Astyanax* cavefish eye development: one is the apoptotic lens, and the other is the small and incomplete retina that lacks a ventral part (the “ventral quadrant”) (Figure 2). From a developmental biology point of view, these two defects seem distinct and independent because the retina and the lens do not share the same embryological origin: the retina is a neural plate (= neural ectoderm/neural tube) derivative, whereas the lens derives from the placodes (= non-neural ectoderm, which gives rise to sensory derivatives of the vertebrate head such as the lateral line, the otic vesicles, or the olfactory epithelium). The two defects however share a common starting point, which is an enlarged expression of *Hedgehog* at the embryonic midline during gastrulation in cavefish [61]. In addition, a 2 h earlier heterochronic expression of *Fgf8* at the anterior midline of cavefish might play an important role as well [62].

### Lens death

Yamamoto and Jeffery have shown that lens apoptosis is the triggering event for subsequent eye degeneration in *Astyanax* cavefish [56]. Indeed, transplantation of a surface lens into a cavefish eyecup morphologically rescues cavefish eye development, whereas transplantation of a cavefish lens into a surface fish eyecup induces the degeneration of the surface fish eye. They have also found that cavefish lens apoptosis is an indirect consequence of increased Hedgehog midline signaling [61]. In support of this, surface fish embryos overexpressing *Hedgehog* mRNA later show apoptotic lens and degenerate eyes. The exact mechanism by which Hedgehog exerts its harmful influence on the lens is unknown, yet it is documented from other model species that early placodal development is largely orchestrated by and dependent on embryonic Hedgehog, Fgf, Wnt and Bmp signaling systems [63-66].

In cavefish, the *Hedgehog* heterotopy (and possibly the *Fgf8* heterochrony) has negative consequences on lens survival, but on the other hand it exerts what appears to be a positive influence on the development of other sensory structures that are enhanced on the cavefish head, such as, for example, taste buds [67]. *Hedgehog* expression is expanded in the oral epithelium of cavefish where it positively influences the size of the jaws and the number of taste buds on the lips [68]. In addition and importantly, there is an inverse relationship between these oral traits and eye development that is pleiotropically linked to Hedgehog signaling, suggesting the possibility of a trade-off between eye loss and oral gain.

Another indication for sensory compensation comes from the recent work of Yoshizawa and colleagues [69]. They have described in cavefish an adaptive vibration attraction behavior, called VAB, which helps them locate food droppings onto the water surface [70,71]. Using quantitative genetic analyses, they showed that the three traits corresponding to the eye size, number of neuromasts in the suborbital region, and presence of the VAB mediated by these neuromasts map together on the same large chromosomal region–but this genomic region is actually much too large for the same gene to pleiotropically control the three traits [69,72,73]. Moreover, in this case, experimental induction of eye regression in surface fish via *Hedgehog* overexpression was insufficient to increase the number of orbital neuromasts or to promote the appearance of VAB [69].

**Figure 3:**
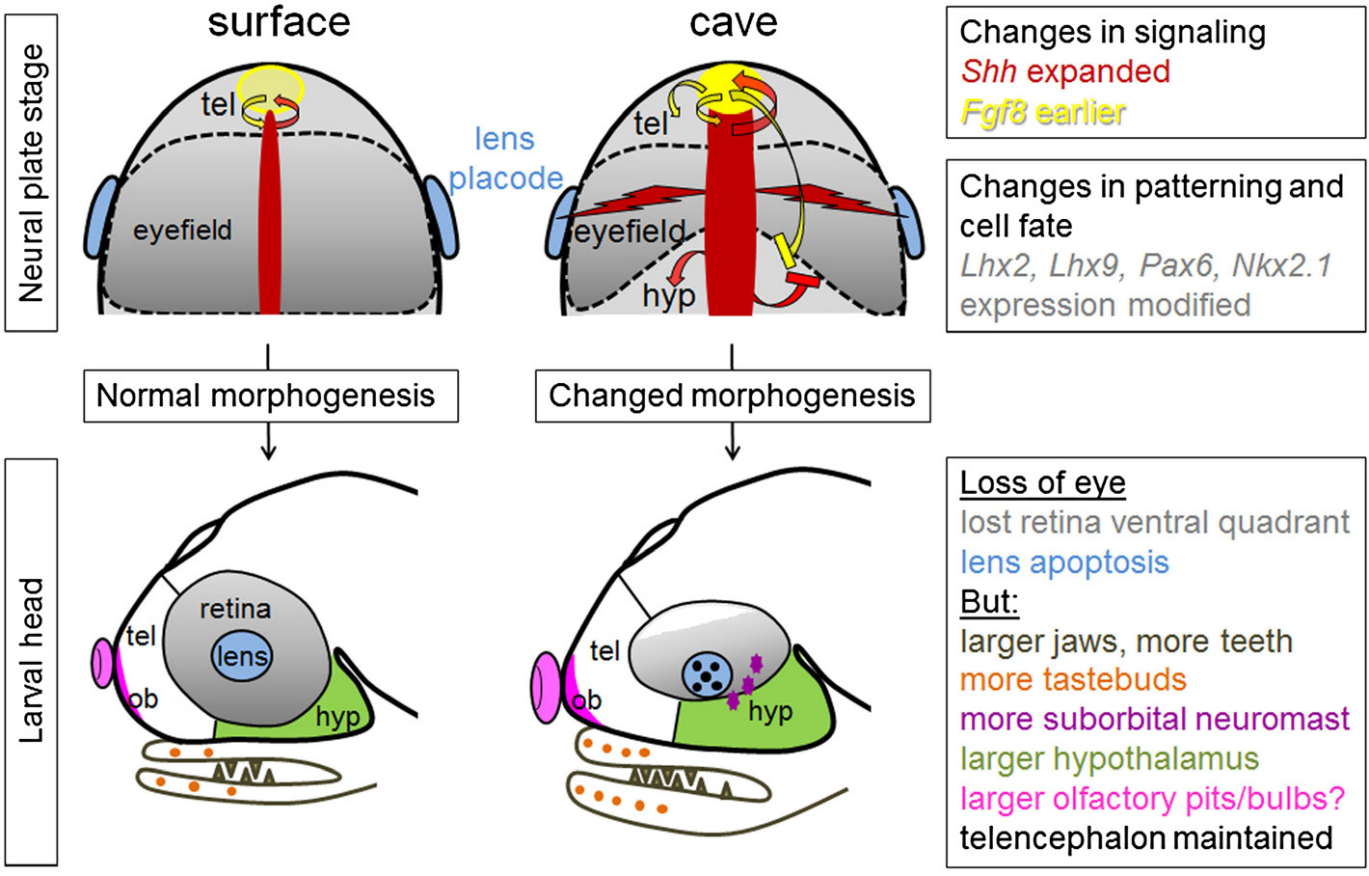
**Embryonic origins of morphological changes in cavefish: loss of eyes and amplification of other neurosensory adaptive structures. Top** Drawings in dorsal views of embryos at neural plate stage in surface fish (*left*) and cavefish (*right*). Signaling molecules are in *red* (Hedgehog, Fgf8), the anterior neural plate fated to become the forebrain is *grey*, the eyefield (i.e., the portion of the neural plate that will give rise to the retina of the eye) is outlined, and the lens placode is *blue*. *Red arrows* indicate stimulatory or inhibitory interactions between signaling centers and their modifications in cavefish. The *red thunderstorm arrows* indicate the negative influence of midline Hedgehog hyper-signaling on the lens. **Bottom** Drawings in lateral views of the larval head and brain, on which all the known changes in size or number of structures are indicated and compared between surface and cave larvae. *tel* telencephalon; *ob* olfactory bulb; *hyp* hypothalamus.

Even more recently, evidence for *Astyanax* cavefish having larger olfactory pits and higher chemosensory capabilities than their surface conspecifics in the Rio Subterráneo cave was reported [74]. Taken together, these data suggest multiple developmental changes in *Astyanax* sensory systems: vision (dispensable in the dark) is “compensated for” by other mechano-sensory modalities with adaptive value (to find food in the dark) (Figure 3).

### Small retina

In the cavefish gastrula and neurula, Hedgehog signaling is expanded in the notochord and prechordal plate underlying the neural plate and tube, and in the antero-ventral aspect of the neural tube itself, corresponding to the presumptive territory of the hypothalamus [62]. Such an increase in Hedgehog morphogen signaling modifies the patterning and the fate map of the cavefish neural plate: some cells located in the anterior medial aspect of the cavefish neural plate—under direct influence of Hedgehog—adopt a hypothalamic fate instead of contributing to the ventral part of the retina as they do in surface fish embryos [62]. It is actually possible to rescue this ventral quadrant after pharmacological manipulation of cavefish embryos that reduce Fgf and Hedgehog signaling. These data explain how the ventral quadrant of the retina is missing in cavefish embryos. In fact, this defect appears like a relatively mild morphogenetic variation that would not have major consequences on cavefish visual development if the lens on the other end did not enter apoptosis and trigger eye degeneration. This notion of a slightly reduced but quite healthy cavefish retina tissue is supported by two types of evidence. First, when a surface fish lens is transplanted into a cavefish retina, the host retina develops and one obtains a cavefish with an eye [56], even if it is doubtful that this eye is functional ([75] and see below). Second, while the cavefish retinal cells die as they are generated because of the lack of lens-derived (and pigmented epithelium-derived) survival signal(s), the genetic programs for retinal neurogenesis, layer formation, and establishment of connectivity appear relatively intact [53,57,76].

As one could anticipate, increased Hedgehog signaling impacts on other aspects of cavefish neural plate and forebrain development: the migration of interneurons from the ventral telencephalon to the olfactory bulbs is increased [77], the hypothalamic anlage proliferates more and is larger in cavefish than in surface fish [62,77], and the size of specific neuronal groups such as serotonergic neurons in the anterior hypothalamus is enlarged [78]. The latter modification has important functional consequence(s), as it increases foraging in cavefish, a behavior that may be considered adaptive to survive in the dark [78,79]. On the other hand, Hedgehog hyper-signaling is “compensated” for in cavefish by earlier expression of *Fgf8* at the antero-dorsal extremity of the neural tube [62], a phenomenon that is thought to counteract the ventralizing effects of Hedgehog and allow for a properly organized brain to develop. Although this has not yet been investigated directly, such a modulation of Fgf8 signaling probably has important consequences on the development of pallial regions of the cavefish forebrain, which include the olfactory bulbs [80,81] (Figure 3).

#### ***Conclusions: selection and constraints***

In sum, the current data available on the developmental mechanisms of eye degeneration in *Astyanax* cavefish indicate a role of pleiotropic factors. These pleiotropic factors would, on the one hand, control eye size and degeneration, but on the other hand they would favor developmental changes responsible for adaptive changes, including changes in sensory and neuromodulatory systems. This points to indirect selection as an underlying evolutionary mechanism. It should be noted, though, that the “developmental Hedgehog hypothesis” does not have a genetic correlate: the genomic regions encompassing Hedgehog genes are not among those that contain the 12 QTLs (quantitative trait loci) identified for the control of eye and lens size in Pachón cavefish [43]. Other factors, possibly upstream of Hedgehog, must be involved.

Some authors have proposed a modular view of cavefish development [82], some modules being lost (the eye and pigmentation modules) and some being expanded (the taste bud and neuromast modules), and they suggested that cavefish represents an illuminating example of module interaction, uncoupling of modules, and module expansion. More recently, Wilkens discussed this idea with a genetic view, i.e., the absence of phenotypic correlations on F2 hybrids between cavefish and surface fish, which suggests that the different regressive and constructive modules are not correlated and do not show genetic linkage [83]. He proposed that “the ‘subordinate genes’ of the module-specific gene cascades (including Hedgehog genes) may be expressed in developmental pathways of quite different modules. Thus, a cave fish organism would consist of a set of modules, which evolve independently.”

Finally, it is worth mentioning that Hedgehog expression is also affected in the cave amphipod *Gammarus minus*[84]. Arthropod eye development is totally different from vertebrates, but still controlled by Hedgehog, which, as shown in Drosophila, regulates both proliferation and differentiation [85]. Contrarily to cavefish, Hedgehog expression is decreased in the cave amphipod [84], and this may relate to the type of control exerted by Hedgehog on the addition of ommatidiae at the margin of the compound eye. It is nevertheless a striking coincidence that the regulation of Hedgehog expression is affected in cave animals belonging to both vertebrate and arthropod phyla. It points to the multiple and powerful effects of Hedgehog molecules as morphogens and to their critical influence in the matter of morphological evolution [86].

Another point is worth discussing to conclude this section: why do cavefish first develop eyes, before undergoing a progressive degeneration process? It looks like an unnecessarily complex and energetically costly developmental pathway. We think that such a process is the consequence of a strong developmental constraint at early stages of forebrain development in vertebrates. In fact, at the neural plate stage, the cells fated to give rise to the retina and the rest of the forebrain (telencephalon and diencephalon) are intermingled and not strictly segregated [87,88]. Moreover, the morphogenetic cell movements for the formation of the optic vesicles and for the so-called “subduction” of the hypothalamus under the retina field and telencephalic primordium are intimately linked [89]. Thus, we suggest that it is an absolute requirement for a vertebrate neural plate and tube to undergo these coordinated movements, including the evagination of the optic vesicles; otherwise, the entire forebrain would be malformed and the embryo would not be viable. Cavefish embryos therefore nicely illustrate an example of an absolute developmental constraint on morphological evolution.

### The loss of eyes: insights from transcriptomics and genomics

Molecular evolution data are still sparse in the field of cave animals, but things are changing rapidly because of the advent of new sequencing technologies.

### Naked mole rat (*Heterocephalus glaber*)

The publication of the naked mole rat genome has brought some first insights in this respect [90]. Of note, it is not strictly a cave animal, but lives underground in burrows, in the absence of light. Interestingly, among inactivated or missing genes in this genome, there is enrichment in functional categories corresponding to olfactory receptor activity and visual perception. Indeed, out of 200 genes categorized with the GO (gene ontology) term “visual perception,” 10% are pseudogenes showing insertion or deletion events. These include two crystallins (cryBA4 and cryBB3), two out the four vertebrate opsins, and other genes involved in phototransduction and photoreceptor function. In addition, cryGS carries a point mutation. For several of these genes, including the three cited crystallins, a relaxation of functional constraints was noted, as seen through the ratio of non-synonymous to synonymous substitutions. Thus, it seems that genes involved in visual function have been particularly targeted by loss-of-function mutations during the evolution of the naked mole rat genome, suggesting neutral evolution through genetic drift.

Although this is not directly related to the loss of eyes in this underground animal, it is noteworthy that genes under positive selection in its genome include several genes involved in telomere shaping, protection, and regulation [90]. This is particularly interesting to relate to the exceptional longevity of the naked mole rat (32 years) [91], a feature that is remarkably shared by at least another cave-living species, the cave salamander *Proteus* (over 100 years) [92].

### *Astyanax* cavefish

In *Astyanax* cavefish, for which the genome is not fully available yet but soon should be (http://www.ncbi.nlm.nih.gov/assembly/GCA_000372685.1/#/def), some genes that sounded attractive to explain the loss of vision were investigated in a “candidate gene approach.” Disappointingly, the *Pax6* sequence was found identical in cavefish and surface fish [93]. More encouraging, opsin genes (one red and two green) were found to accumulate high rates of nucleotide substitutions and C to T transitions in cavefish, features that are considered a sign of pseudogene formation [94]. Interestingly in this study, the number of accumulated mutations was not correlated to the “age” of the cavefish population, which can be estimated using the degree of troglomorphy and a combination of molecular phylogenies and population genetics approaches [15,32,39,95]. More recently, a large-scale survey of polymorphism and fixed mutations in the transcriptome of a surface and a cave population of *Astyanax* revealed that a high proportion of the genes carrying mutations responsible for radical amino-acid changes in the cavefish lineage correspond to “eye genes,” as deduced from their strong and specific expression in the zebrafish developing visual system [96]. Therefore, in cavefish also, eye-related genes appear to be under relaxed selection.

Such a phenomenon was also recently reported for rhodopsin in amblyopsid cavefishes [97]. Their visual pigment independently accumulated unique loss-of-function mutations in at least three cave lineages over the last 10.3 Ma. In addition, for those cave lineages that still possess functional rhodopsin, they exhibit increased rates of non-synonymous mutations that have greater effect on the structure and function of rhodopsin compared to those in surface lineages, suggesting that these mutation accumulate as a result of a loss of functional constraint [97].

### Regulatory genome

The reports cited above only concern the evolution of the coding sequences. However, phenotypic evolution (including the loss of structures) can also occur through changes in non-coding, *cis*-regulatory sequences. Famous examples include the loss of the pelvic spine in freshwater sticklebacks through deletion of a *Pitx1* enhancer [98,99], or gain or loss of pigmentation patterns in Drosophilae through co-option or mutation of regulatory elements in the pigmentation gene *yellow*[100]. Although the exact mechanism is unknown, this happened for crystallin αA in cave *Astyanax*[55,101]. This chaperone and anti-apoptotic crystallin whose coding sequence is almost identical in surface fish and cavefish (one amino-acid difference only) is strongly downregulated in the cavefish lens during development and was suggested as a potential major player in the onset of cavefish lens apoptosis. In the naked mole rat *Heterocephalus glaber*, gamma-crystallins are turned off after birth [46]. In the mole rat *Spalax ehrenbergi*, the αB-crystallin promoter and intergenic regions have selectively lost lens activity after 13.5 days of embryogenesis [102,103]. These examples show that changes in regulatory sequences also occurred in cave and other underground animals.

### Conclusions on molecular evolution data

In the two species for which large-scale molecular evolution data are available, the results converge to show an over-representation of “eye genes” in those that are affected by loss of function or radical amino acid substitutions [90,96]. In both species, the lens seems to play a central role in eye degeneration [46,56], and lens crystallins seem particularly targeted by substitutions in coding and in non-coding regions. On the retinal side, genes involved in photoreception also accumulate substitutions. We think that such enrichment for mutations in eye-related genes supports the hypothesis that these genes accumulate mutations as a result of relaxed selection on visual system genes in caves and therefore suggests a genetic drift mechanism. Interestingly, Wilkens, discussing the rather large phenotypic variability observed in cavefishes between individuals in a given population as well as between populations, also attributed this variability of regressive traits to relaxed selection [83]. Alternatively, loss of function mutations may be under positive selection in the cave environment precisely because they lead to a reduced eye size. However, there are 12 QTLs controlling eye size, and probably much more radical mutations accumulated in cavefish. For this reason, we propose that some of the initial substitutions that occurred in cavefish eye genes may have been selected—a mechanism that is supported by the consistent negative polarities of eye QTLs [43] —but that some other subsequent substitutions can be the result of genetic drift.

As stated above, the transplantation of a surface fish lens into a cavefish optic cup rescues an eye structure in cavefish, and a positive effect of the lens on retinal cell survival was demonstrated, including on rod photoreceptors [56,57]. However, in cavefish, photoreceptor outer segments never form, opsins are only very transiently expressed [52], and evidence is accumulating to suggest that a number of genes involved in retinal development and function are knocked down. This probably explains why transplanted cavefish with an eye do not see despite rescued optic morphology [75]. In fact, in this paper Romero et al. [75] proposed that “regressive changes have evolved beyond the level of the eye in the cavefish visual system.” Molecular data are now supporting this hypothesis well.

## Conclusion

Theoretically, and although they have been much debated and opposed to each other, the neutral and selective hypotheses for the loss of eyes in cave animals are not mutually exclusive processes. Evidence from developmental biology and from molecular evolution studies suggests that both indeed occurred together.

Many questions remain. What are the exact developmental constraints on morphological evolution in early embryos [62]? Which genes correspond to the 12 QTLs associated with “eye size” in *Astyanax* cavefish [104]? Are some of them really pleiotropic developmental genes [83,105]? Are genetic linkage and QTL clusters controlling the concerted evolution of multiple traits in cave animals [69,104]? Can we get independent clues about adaptive selection through the identification of a selective sweep at some loci? What happened first, selection or drift, when surface-type ancestors were trapped into caves? Were some pre-existing and necessary mutations present at low frequencies in the surface populations, allowing un-delayed selection and rapid adaptation for the great survival and reproductive challenges associated with the cave environment? These questions may get some answers from integrative analyses relying on multiple evo-devo approaches associated with thorough ecological and population genomic studies.

## Competing interests

The authors declare that they have no competing interests.

## Authors’ contributions

SR and DC wrote the review. Both authors read and approved the final manuscript.
